# Genetic Comparison of ESBL-Producing *Escherichia coli* from Workers and Pigs at Vietnamese Pig Farms

**DOI:** 10.3390/antibiotics10101165

**Published:** 2021-09-25

**Authors:** Duong Thi Quy Truong, Yaovi Mahuton Gildas Hounmanou, Son Thi Thanh Dang, John Elmerdahl Olsen, Giang Thi Huong Truong, Nhat Thi Tran, Flemming Scheutz, Anders Dalsgaard

**Affiliations:** 1 National Institute of Veterinary Research, Hanoi 10000, Vietnam; truongduong2603@gmail.com (D.T.Q.T.); chienson2006@yahoo.com (S.T.T.D.); tgiang.vty83@yahoo.com (G.T.H.T.); trannhatbn1991@gmail.com (N.T.T.); 2 Department of Veterinary and Animal Sciences, University of Copenhagen, 1870 Frederiksberg C, Denmark; jeo@sund.ku.dk; 3 Department of Bacteria, Parasites and Fungi, Statens Serum Institute, 2300 Copenhagen S, Denmark; fsc@ssi.dk

**Keywords:** ESBL, genomics, commensal *E. coli*, one health

## Abstract

We analyzed and compared genomes of Extended Spectrum Beta-Lactamase (ESBL)-producing *Escherichia coli* from pigs and pig farm workers at 116 farms in Vietnam. Analyses revealed the presence of *bla*_CTX-M-55_, *bla*_CTX-M-27_, *bla*_CTX-M-15_, *bla*_CTX-M-14_, *bla*_CTX-M-3_, *bla*_CTX-M-65_, *bla_CTX-M-24_*, *bla_DHA-1_*, and *bla_CMY2_* in both hosts. Most strains from pigs contained quinolones (*qnr*) and colistin resistance genes (*mcr*-1 and *mcr*-3). Isolates predominantly harbored more than one plasmid replicon and some harbored plasmid replicons on the same contigs as the ESBL genes. Five strains from farm workers of ST38 (2), ST69 (1), and ST1722 (2) were classified as either uropathogenic *E. coli* (UPEC_HM_)/extraintestinal pathogenic *E. coli* (ExPEC_JJ_) or UPEC_HM_, and the remaining were genetically distinct commensals. A high heterogeneity was found among the ESBL-producing *E. coli* from pigs and workers, with most isolates belonging to unrelated phylogroups, serogroups, and sequence types with >4046 Single-Nucleotide Polymorphisms-(SNPs). In comparing the genomes of pig isolates to those from humans, it appeared that ESBL-producing *E. coli* in workers did not predominantly originate from pigs but were rather host-specific. Nevertheless, the occurrence of ESBL-producing *E. coli* carrying plasmid-mediated colistin and quinolone resistance genes in pigs could represent a potential source for horizontal transmission to humans through food rather than direct contact.

## 1. Introduction

The World Health Organization (WHO) regards antimicrobial resistance (AMR) as one of the most important threats to public health because of the diminished effectiveness of antimicrobial treatment [1]. Studies have shown that extensive use of antimicrobials in livestock production affects AMR in humans [2,3] and both resistant bacteria in livestock and food should therefore be monitored.

Pork is the main meat consumed by people in Vietnam and, on average, each member of the country’s 95 million people consumes 29 kg pork per year [4]. The pig production is the fifth largest in the world with 2386 metric tons of pork meat [5]. Production is mainly carried out on small-scale farms with a low level of biosecurity and hygiene, as well with frequent use of antimicrobials for disease prevention and treatment, and as growth promoters in commercial feed [6]. Thus, it has been estimated that, on average, 287 to 564 mg active antimicrobials are used per kilogram of live pig produced in Vietnam [6,7].

Antimicrobials of critical importance in human medicine such as colistin [8] are used for prophylaxis and treatment of pigs in Vietnam [9]. Increasing levels of β-lactams, including cephalosporin, and colistin resistance have been reported as, e.g., 100% of *E. coli* isolated from pigs were resistant to ampicillin and 40% of isolates were resistant to colistin [9,10]. Dang et al. [10] found that 89% of *E. coli* from pigs in small and medium-scale farms in Northern Vietnam were resistant to cefotaxime, a third generation cephalosporin. A high level of resistance to gentamycin, ciprofloxacin, and third generation cephalosporins was also found on chicken farms in Southern Vietnam [11].

High levels of bacterial antimicrobial resistance is not only found in livestock but also in the food and people in Vietnam [12]. It is increasingly suggested that production animals are important sources of the antimicrobial resistant bacteria in farm workers. While it is well-documented that resistant bacteria can be transmitted through the food chain, there is a paucity of evidence that direct contact is a main route of transfer for enteric resistant bacteria, including ESBL-producing *E. coli*, from animals to farmers [13,14,15,16]. The present study aims to determine and compare the genetic characteristics of ESBL-producing *E. coli* from pigs and pig farm workers. To address this aim, we used whole-genome sequencing (WGS) to analyze ESBL-producing *E. coli* isolated from fecal samples of pigs and pig farmers in Bac Ninh Province in Northern Vietnam.

## 2. Results

### 2.1. Antimicrobial Resistance in E. coli Isolated from Pigs and Farm Workers

*E. coli* was isolated from MacConkey agar without cefotaxime from all pig fecal samples (116), while only 94 fecal samples from farm workers yielded *E. coli*. The susceptibility to antimicrobials was determined for one randomly picked and confirmed *E. coli* isolate per fecal sample (Table 1). The results showed that the resistance levels in pigs were slightly higher than in farm workers. For instance, resistance to ampicillin (AMP), sulfonamide (SUL), trimethoprim (TMP), and streptomycin (STR) was present in 86% (100/116), 82% (96/116), 72% (84/116), and 56% (65/116) of the pig isolates, respectively, compared to 72% (68/94), 67% (63/94), 65% (61/94), and 42% (40/94) of the isolates from farm workers. There was a significant difference of resistance levels to AMP (*p* = 0.02) and SUL (*p* = 0.01) in isolates from pigs and farm workers. Resistance to colistin (COL) in the pig isolates was 38% (44/116), which was much higher (*p* = 0.003) than in the human isolates (15%; 14/94). Resistance to cephalosporin drugs, including ceftiofur (EFT), ceftriaxone (CRO), and cefoxitin (FOX), was, however, higher in the isolates from the workers than in the pig isolates (Table 1). Three isolates from pigs and seven isolates from workers were fully susceptible to all tested antimicrobials. Sixty-two out of 94 human isolates (66%) were classified as multi-drug resistant (MDR), while 96 isolates were MDR among the 116 pig isolates (82%). A total of 55 different AMR profiles were observed among the 116 pig *E. coli* isolates, while 50 profiles were found among the isolates from the farm workers (Table S1). Twenty-two profiles were shared between pig and human isolates, accounting for 59% of the total isolates.

### 2.2. Occurrence of ESBL-Producing E. coli in Pigs and Pig Farm Workers

**Figure 1:**
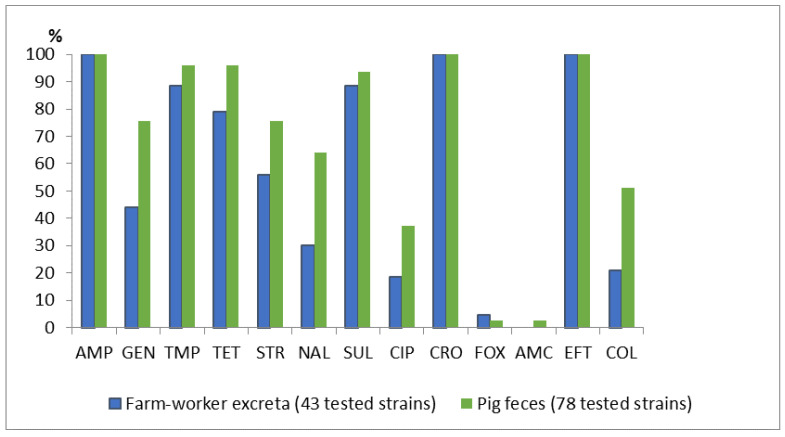
Antimicrobial resistance of ESBL-producing *E. coli* from pigs and pig farm workers. Abbreviations: AMP, Ampicillin; GEN, Gentamycin; TMP, Trimethoprim; TET, Tetracycline; STR, Streptomycin; NAL, Nalidixic acid; SUL, Sulphonamide; CIP, Ciprofloxacin; CRO, Ceftriaxone; FOX, Cefoxitin; AMC, Amoxicillin-clavulanic acid; EFT, Ceftiofur; and COL, Colistin.

Fifty-eight of the 94 (62%) human samples and 87/116 (75%) of the pig samples yielded presumptive *E. coli* colonies on MacConkey agar containing 2 mg/L cefotaxime. One isolate from each sample confirmed as *E. coli* in the phenotypic testing was picked and tested for the ESBL phenotype. A total of 43/58 (74%) isolates from workers and 78/87 (90%) of the pig isolates were confirmed as ESBL-producing *E. coli* by the double-disk synergy test. These ESBL strains were all subjected to further antimicrobial resistance testing, in which all the isolates were found resistant to AMP, CRO, and EFT (Figure 1). Co-occurrence of resistance to colistin was observed in 51% of the pig isolates (40/78) and 21% of the human isolates (9/43). Forty-nine resistance profiles were found among the 121 ESBL-producing *E. coli* (Table S2), of which 11 profiles were shared between the human and pig isolates.

### 2.3. Antimicrobial Resistance Genes in ESBL-Producing E. coli

#### 2.3.1. ESBL Genes

**Figure 2:**
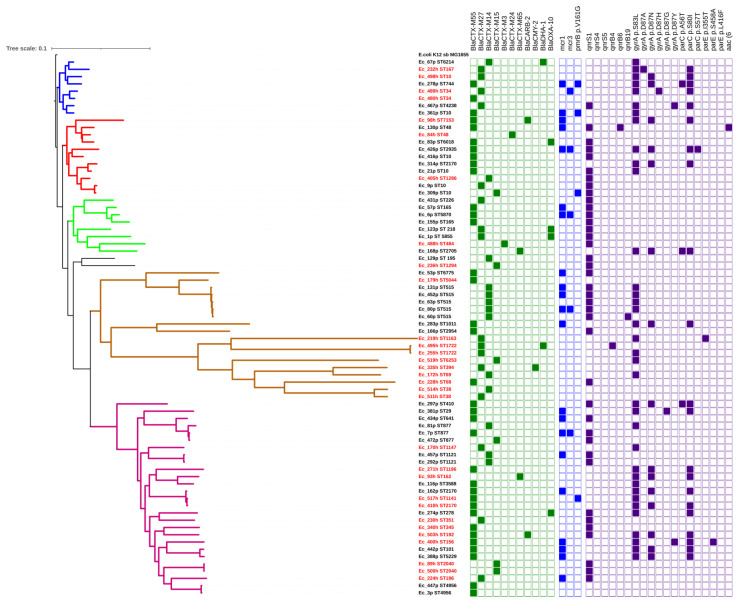
Phylogeny of ESBL-producing *E. coli* from pigs and pig farm workers. The isolates from workers are shown with red font node labels and the pig isolates are in black. The squares to the right indicate gene presence (full square) and absence (empty square). Note: green, ESBL genes; blue, plasmid-mediated colistin resistance genes and mutations; and purple, quinolone resistance (plasmid-mediated genes and point mutations). The color distinctions of the tree branches distinguish six main clusters in which the 74 isolates were regrouped.

Seventy-four ESBL isolates were selected for WGS analysis (see Materials and Methods for selection strategy). The ESBL genes detected in the 43 pig isolates were *bla*_CTX-M-55_ (22/43), *bla*_CTX-M-14_ (10/43), *bla*_CTX-M-27_ (5/43), *bla*_OXA-10_ (4/43), *bla*_CTX-15_ (2/43), and *bla*_CTX-M-65_ (1/43) (Figure 2). Two of the isolates harboring *bla*_OXA-10_ also carried *bla*_CTX-M-55_, while the other two isolates contained *bla*_CTX-M-27_. One isolate (Ec-67 from farm E-04) harboring *bla*_CTX-M-14_ co-carried an *Amp*C gene (*bla*_DHA-1_). The ESBL genes detected in the human isolates included *bla*_CTX-M-27_ (11/31), *bla*_CTX-M-55_ (10/31), *bla*_CTX-15_ (4/31), and *bla*_CTX-M-14_ (3/31). The *bla*_CARB-2_ gene was found in two isolates also carrying *bla*_CTX-M-55_. The *bla*_CTX-M-3_, *bla*_CTX-M-24_, and *bla*_CTX-M-65_ genes were found in three human isolates. Two isolates from farm workers carrying *bla*_CTX-M-27_ were also shown to harbor *Amp*C genes; one isolate carried *bla*_DHA-1_ and another isolate had *bla*_CMY-2_. Two isolates from workers harbored two *bla* genes, i.e., *bla*_CTX-M-55_ and *bla*_CARB-2_ (Figure 2). Overall, *bla_CTX-M_* genes were the most dominant of ESBL genes in all the samples from pigs and pig farm workers. Two AmpC genes, namely *bla*_DHA-1_ and *bla*_CMY-2_, were always co-occurring with other *bla* genes.

#### 2.3.2. Colistin Resistance Genotypes

Eighteen of the 43 ESBL-producing *E. coli* isolates from pigs (four with *bla*_CTX-M-14_ and 14 with *bla*_CTX-M-55_) carried colistin resistance genes. All these 18 isolates carried the *mcr*-1 gene, of which four isolates also contained *mcr*-3. Moreover, three of those having only *mcr*-1 revealed a mutation on the *pmr*B gene at position p.V161G (Valine- > Glycin), encoding colistin resistance (Figure 2). However, one isolate (Ec_309) had only the *pmr*B mutation and was susceptible to colistin in the phenotypic test. Among the isolates from farm workers, six isolates were found to be colistin-resistant in the MIC test. Three isolates (two isolates harboring *bla*_CTX-M-55_ and one isolate harboring *bla*_CTX-M-27_) carried the *mcr*-1 gene, while one isolate (Ec489 with *bla*_CTX-M-27_) carried only the *mcr*-3 gene. The two other isolates (Ec_219-Farm A10 and Ec_255-farm E05) showing colistin-resistant phenotypes did not carry a resistance gene or known mutation that supports such resistance. Overall, the presence of *mcr*-1 and *mcr*-3 together with mutations in the *pmr*B gene were the main genotypes associated with colistin resistance in the analyzed ESBL-producing *E. coli* genomes and were encountered more in the pig rather than human isolates.

#### 2.3.3. Quinolone Resistance

The genes *qnr*B4, *qnr*B6, *qnr*B19, *qnr*S1, *qnr*S4, and *qnr*S5, belonging to the plasmid-mediated quinolone resistance category (PMQR) were found in one or more of the 74 ESBL-producing *E. coli*. The *qnr*S1 gene was the most commonly found and was detected in 10 human isolates and 29 pig isolates. Some isolates carried more than one PMQR gene, e.g., the pig isolate Ec60-farm E01 co-carried *qnr*S1 and *qnr*B19 genes, and the pig isolate Ec_130-farm B09 carried *qnr*B6 and *qnr*S1 (Figure 2). Mutations in the quinolone resistance-determining region (QRDR) of topoisomerase genes were found in 51% (22/43) of the pig isolates and in 17 of the human isolates, resulting in amino acid substitutions in the *gyrA* as well as in *parC* and *par*E (Table 2).

Three human isolates harboring the *qnr*S1 gene were phenotypically susceptible to NAL and CIP. Among pig isolates, 13 isolates harboring the *qnr*S1 gene also were phenotypically susceptible, while one isolate showing resistance to nalidixic acid had no resistance genotype. In summary, 18/43 ESBL-producing *E. coli* from pigs co-carried colistin and quinolone resistance genotypes (Figure 2), but four of these isolates did not show phenotypic resistance to quinolones. Four of the 31 human ESBL-producing isolates had resistance genotypes for colistin and quinolone, but one of the four (Ec_224) was phenotypically susceptible to quinolones.

#### 2.3.4. Resistance Genotypes to Other Antimicrobials

Twenty aminoglycoside resistance genes were found in the 74 ESBL-producing *E. coli* isolates, of which 19 genes were present in pig isolates. The most common aminoglycoside resistance genes in the pig isolates were *aad*A1 (28), *aph* (3′-Ia (21), *aph* (6)-Id (21), *aac* (3)-IId (20), and *aad*A2 (17) (Table S3). Two pig isolates (Ec_168 and Ec_447) carrying one or two aminoglycoside resistance genes (*aad*A1 and/or *aad*A2) were susceptible to GEN and STR. The isolates from workers carried 11 aminoglycoside resistance genes, mainly *aph* (6)Id (12), *aph* (3′-Ib (11), and *aad*A1 (10) (Table S3). Four of them carried only one or two of the genes (*aad*A2 and/or *aad*A5) but were susceptible to GEN and STR, while one isolate (Ec_236) was resistant to STR but did not carry a resistance gene.

Analysis of the genetic background of resistance to sulfonamides revealed that *sul*1, *sul*2, and *sul*3 genes were present in both the human and pig isolates. The pig isolates mostly harbored *sul3* (28), while *sul*1 was predominant in the isolates from workers (14). One human isolate (Ec_480) showed phenotypic sulphonamide resistance but did not contain any associated resistance gene. Trimethoprim resistance genes were found in 42 pig isolates and 23 human isolates. Genes detected included *dfr*A1, *dfr*A12, *dfr*A14, *dfr*A15, *dfr*A16, *dfr*A17, and *dfr*A27. Six of these seven genes were found in the pig isolates with the dominant genes being *dfr*A12 (*n* = 20) and *dfr*A14 (*n* = 19). Five genes were present in isolates from workers, particularly *dfr*A17 (*n* = 9) and *dfr*A14 (*n* = 8). One pig isolate (Ec_388) and two human isolates (Ec_179 and Ec_368) showed phenotypic resistance to trimethoprim but no associated gene was detected (Table S3). Tetracycline resistance genes *tet* (A), *tet* (B), *tet* (C), *tet* (M), and *tet* (X4) were found in 42 ESBL-producing *E. coli* from pigs and 22 human isolates (Table S3). *tet* (A) and *tet* (M) were the most dominant genes in both the pig and human isolates. One pig isolate (Ec_9) and one human isolate (Ec_219) were resistant to tetracycline but did not carry any *tet* genes, whereas the human isolate Ec_489 carrying *tet* (M) was phenotypically sensitive to tetracycline. Moreover, 37 of the pig isolates and 14 isolates from the workers carried chloramphenicol resistance genes, mainly *flo*R and *cml*A1 (Table S3), with *flo*R present in 33 pig isolates and 10 human isolates, while *cml*A1 was found in 24 pig isolates and five human isolates. Macrolide resistance genes detected were *erm* (B), *lnu* (F), *lnu* (G), *mdf* (A), *mef* (B), and *mph* (A), of which the *mdf* (A) gene was present in all the 74 ESBL-producing *E. coli* isolates. Fosfomycin and rifampicin resistance genes were only found in a few pig isolates, with three isolates carrying the fosfomycin resistance gene *fos*A3. The rifampicin resistance genes *aar*2 and *aar*3 were present in seven and two pig isolates, respectively (Table S3).

Metal resistance genes encoding resistance to copper, zinc, cobalt, and cadmium were found in all 74 ESBL-producing *E. coli* isolates (Table S5). Two pig isolates and four human isolates carried the metal resistance genes on plasmid contigs. Five pig isolates and ten human isolates carried the chromate transport protein *ChrA* on plasmids, encoding chromium resistance. Mercury resistance genes were found in 42 out of 43 pig isolates and 27 out of 31 human isolates, of which six pig isolates and two human isolates carried mercury resistance operon (*merC*/*merE*/*merT*) on plasmids.

### 2.4. Plasmid Replicons in ESBL-Producing E. coli

The isolates had a wide variation and number of plasmid replicons, with 23 plasmid replicons found in 39 pig isolates, ranging from one to six replicons per isolate (Table 3). The most dominant plasmid replicon in pig isolates was IncX1 types (*n* = 8). IncFIA (HI1), IncFIB (K), and IncY co-occurred in four isolates and the IncFII, IncFIB (AP001918), IncFIC (FII), IncN, and p0111 replicons occurred together in three isolates. Only three isolates out of the 43 isolates from pigs (7%) were found to encode plasmid replicons from the same contigs as the ESBL genes. Of these, two isolates harbored *bla*_CTX-M-55_ on the same contig as IncX1 and one isolate harbored *bla*_CTX-M-55_ on the same contig as the IncX2 replicon.

In the 31 ESBL-producing *E. coli* from human feces, 29 harbored plasmid replicons, varying from one to seven replicons per strain. The most frequent replicons were IncFIB (AP001918) (*n* = 12), IncFII (*n* = 6), IncFIA (*n* = 5), IncFIA (HI1) (*n* = 5), IncFIB (K) (*n* = 5), IncFII (pCoo) (*n* = 4), and IncY (*n* = 4). Three of these isolates harbored ESBL genes on the same contigs as the plasmid replicons, including Ec_230 (*bla*_CTX-M-27_ with IncFIA (HI1)), Ec_ 84 (*bla*_CTX-M-24_ with IncP1), and Ec_271 (*bla*_CTX-M-55_ with IncFIB (AP001918)), as shown in Table 2. Our data indicate that *bla*_CTX-M-55_ is likely associated with the IncX (1/2) plasmid replicon types seen in pigs and humans, although this needs to be confirmed in additional isolates.

### 2.5. Genetic Diversity of ESBL-Producing E. coli

The MLST analysis revealed 53 different sequence types among the 74 ESBL-producing *E. coli*. The sequence types were also different between the pig and human isolates from the same farms. The main STs in the pig isolates were ST10 and ST515, with five isolates in each (Table 3). ST10 isolates belonged to phylogroup A and carried the ESBL genes *bla*_CTX-M-55_ (3), *bla*_CTX-M-27_ (1), and *bla*_CTX-M 15_ (1). The isolates of ST515 belonged to phylogroup B1 and serotype H12 (undefined O), and also carried *bla*_CTX-M-14_. Four of the five ST515 isolates had the same O group (O128). Three ST877 isolates (phylogroup B1) carried *bla*_CTX-M-55_, *bla*_CTX-M-24_, or *bla_CTX-M-15_* but originated from three different districts in the Bac Ninh Province. Two isolates belonged to ST1121 and both carried *bla*_CTX-M-14_. The 31 isolates from workers revealed 27 different sequence types. Sequence types ST1722, ST2040, ST34, and ST38 were found in two isolates each. 

Only three sequence types (ST10, ST48, and ST2170) were shared between the pig and human isolates, but the isolates displayed different resistotypes (Figure 2). 

In general, our data revealed that there was high heterogeneity among the ESBL-producing *E. coli* from the pigs and workers with respect to phylogroups, serotypes, and sequence types. In a simmer manner, the phylogenetic analysis also revealed a wide variation with up to 4046 SNPs between the 74 strains (Figure 2, Table S4). Six heterogeneous clades of different sequence types and serotypes appeared with more than 1000 SNPs between the isolates. There was no significant difference of distribution between the pig and human isolates in each cluster (*p* > 0.05). Although each of the clusters contained mixed strains, i.e., human and pig isolates, the strains did not often belong to the same STs, as described above, and they also showed differential status in the resistance genes; for instance, pig isolates harbored *mcr* genes, which were lacking in the human isolates (Figure 2).

Some STs appeared to be genetically narrow, associated to only one of the two hosts in the current study. For example, strains of ST515 from different pig samples only differed by 1 to 3 SNPs and this ST was only found in samples from pigs. In a similar manner, the isolates which belonged to ST1722 only had 10 SNPs between them and were only found in human samples. The human isolates of ST2040 (last cluster in Figure 2) were identical and pig isolates of both ST1121 and ST4956 strains were also closely related with 5–6 SNPs.

The human isolates Ec_410 and Ec_517 were closely related to the pig isolate Ec_162, which only differed by 24 SNPs (Figure 2, Table S4). These three strains, however, were isolated from three different farms with no apparent epidemiological association.

### 2.6. Virulence Genes and Serotypes

The most frequent virulence determinants associated with extra-intestinal pathogenic *E. coli* (ExPEC) were as follows, in descending order: *traT* (36), *ompT* (26), *sitA* (24), *fyuA* (19), *irp2* (19), *iutA* (15), *iucC* (15), *hlyF* (13), *chuA* (12), *kpsMII/E* (10), *cvaC* (7), *papA/C* (6), *hra* (4), *afaA/B/C/D/E* (2), *yfcV* (2), *cib* (2), *etsC* (2), and *neuC* (2). All these genes were evenly distributed between the human and porcine isolates except for the UPEC_HM_-related *chuA*, which was found in ten human isolates and two pig isolates, and the ExPEC_JJ_-associated *kpsMII/E*, which was found in eight human isolates and two pig isolates. *hra* was only found in four human isolates (Table 3). Three strains (Ec_172 ^h^, Ec_511 ^h^, and Ec_514 ^h^) could be classified as uropathogenic *E. coli* (UPEC_HM_) and ExPEC_JJ_. Two human isolates (Ec_495 and Ec_255) could be classified as UPEC_HM_. These five isolates were of different serotypes (Table 3). Virulence determinants not associated with ExPEC were found in more than two isolates and were as follows, in descending order: *lpfA* (31), *iss* (26), *astA* (20), *air* (10), *cma* (10), *eilA* (8), *iha* (5), *aap* (5), and *senB* (3). These genes were evenly distributed between isolates of porcine and human origin, except *lpfA* (20 of 31), *iss* (19 of 26), *astA* (12 of 20), and *cma* (7 of 10), which were found more often in the porcine rather than human isolates. *aap* was only found in five human isolates (Table 3). Only one isolate (Ec381 ^p^) of serotype O123/186:H11 harbored the *eae* gene allele (encoding intimin), as well as harbored 13 genes (*astA, cif, efa1, espA, espB, espJ, iha, katP, lpfA, nleA, nleB, nleC*, and *tir*) usually associated with the attaching and effacing of both *E. coli* (AEEC) and ExPEC-related genes *iucC, iutA, ompT, traT, fyuA,* and *irp2*.

## 3. Discussion

ESBL-producing *E. coli* from pigs in Vietnam was found to differ genetically from ESBL *E. coli* obtained from farm workers. A high number of SNPs were detected between the isolates and together with a wide diversity in sequence types, serotypes, phylogroups, resistance genes, and plasmid replicon types, the results suggest a highly heterogeneous population with little evidence of transmission of ESBL-producing *E. coli* between the pigs and workers. Only one ESBL-producing *E. coli* was isolated from each fecal sample and analyzed by WGS, and we cannot rule out that pigs and farmers share strains, which are present in very low concentrations. The virulence gene profiles also suggested that the ESBL-producing *E. coli* analyzed were primarily commensal bacteria, which corroborates findings in a related investigation of pig farms in the same study area [10]. The present study did not compare *E. coli* from pork (food) to *E. coli* from humans and we cannot rule out that some of the human isolates originated from pigs. However, our study seems to rule out that direct contact with pigs at the farm level is a main route of exposure to ESBL *E. coli*. A similar situation was previously documented when *E. coli* from different livestock were shown to be of distinct lineages compared to the isolates collected in humans [17].

Five strains from workers could be classified as either UPEC_HM_/ExPEC_JJ_ or UPEC_HM_. This classification has been derived by comparing limited sets of virulence genes with epidemiological and infection model data, and could indicate extra-intestinal virulence potential in the form of urinary tract infection (UTI) and/or bacteremia [18,19]. We found that the ESBL genes were present in a wide variety of *E. coli* STs and that some STs appeared to be more restricted to isolates as specific hosts [20]. ST69 ExPEC_JJ_ has been isolated from cases of bacteremia in Spain and France [21]. Two of our isolates were ST38, which has been described as an evolving enteroaggregative *E. coli* (EAEC) in the United Kingdom [22], but we did not identify the *aggR* genes defining EAEC. These strains were only found in workers, which could indicate that they are host-specific. Similar to our study, commensal *E. coli* from pigs in Denmark carried ST10 as the most common sequence type [23]. The dominance of the same ST type in pig isolates from Vietnam strains and Europe could suggest that this ST type is widespread in *E. coli* from pigs, although further studies are needed to determine this.

A study of poultry farms in the Red River Delta of Northern Vietnam showed that 83.1% of farm workers and 74.1% of chickens carried ESBL-producing *E. coli* [24], whereas a study of small-scale poultry farms and farming households in Southern Vietnam found that 31.4% of farmers and 14.7% of chickens carried ESBL-producing *E. coli* [16]. ESBL-producing *E. coli* has also been found in 31% of pork meat and 73.5% of asymptomatic resident volunteer workers at a local wholesale market in Central Vietnam [25]. Other studies confirm that the general Vietnamese population seems to have frequent carriage of ESBL-producing *E. coli* among residents in Thai Binh Province in Northern Vietnam, showing a prevalence of 61.2% ESBL-producing *E. coli* [26], while 51% (111/198) of residents in suburban Hanoi harbored ESBL-producing *E. coli* [27]. Our findings confirm that the prevalence of ESBL-producing *E. coli* in people and animals are high in Vietnam.

The ESBL genes found in most of the pig isolates belonged to *bla*_CTX-M_ groups 1 (CTX-M15, CTX-M27, and CTX-M55) and *bla*_CTX-M_ group 9 (CTX-M14 and CTX-M65). This confirms findings from previous studies that *bla*_CTX-M_ group 1 and 9 are predominant ESBL genes in pig farms and in both pork and retail markets [10], as well as in beef, chicken, and fish [13,28,29] in Vietnam. CTX-M55 was the most common gene detected in ESBL-producing *E. coli* from pork sold at wholesale markets in Central Vietnam [25] and this was the most common gene in the pig isolates in our study. All our ESBL-producing *E. coli* isolated from humans carried *bla*_CTX-M_ group 1 and 9, including CTX-M55 (most common), CTX-M27, CTX-M15, CTX-M14, CTX-M24, and CTX-M65; notably, these are genes which were also carried by ESBL-producing *E. coli* isolated from the pork and workers at wholesale markets, from urinary tract infection patients in Central Vietnam [25], and from both poultry and poultry farmers (CTX-M55) [16]. Together these data show that *bla*_CTX-M_ genes are the dominant ESBL genotypes circulating in Vietnam and are widely distributed in humans, livestock, and foods in different parts of the country.

One of the emerging concerns regarding ESBL-producing *E coli* is the co-occurrence of colistin and quinolone resistance, and many of our strains, particularly those from pigs, showed such a resistotype. Colistin is a last-resort drug for the treatment of multidrug-resistant bacteria such as *Acinetobacter* spp. and *Pseudomonas aeruginosa*, and the spread of ESBL-producing bacteria harboring *mcr* genes are of serious concern [30]. Of particular concern, many isolates showed mobile colistin resistance (*mcr*-1 and *mcr*-3) and plasmid-mediated quinolone resistance (*qnr*S1 and *qnr*B4) genotypes. Quinolone and colistin-resistant ESBL-producing *E. coli* have been reported in Japan, e.g., where a CTX-M-27 and CTX-M-14-producing and ciprofloxacin-resistant *E. coli* of the H30 sub-clonal group within ST131 was implicated in a Japanese regional ESBL epidemic [31,32,33]. The high level of quinolone resistance in the ESBL-producing *E. coli* found in both pigs (64.1%) and farm workers (30.2%) is in accordance with the findings in Southern Vietnam, e.g., where 42.7% to 62.7% ESBL/*Amp*C *E. coli* were resistant to quinolones [28,29]. Our results suggest that one mutation and presence of a *qnr* gene could be sufficient to produce phenotypic quinolone resistance, which is in agreement with previous studies of ESBL-producing *E. coli* [31,34]. Quinolones (enrofloxacin and norfloxacin) are frequently used for the prevention and treatment of diarrhea in piglets in Vietnam [35]. It has been reported that quinolone-resistant *E. coli* have spread before the acquisition of ESBL genes [36]. This could explain the high level of quinolone resistance in the ESBL-producing *E. coli* found in our study. Our sequence data did not allow for concluding that *mcr* genes, along with quinolone and ESBL genes, were located on the same contigs as the plasmid replicons most likely because of the short reads-sequencing platform used. Nevertheless, the contigs harboring each of these genes yielded only plasmid hits in a preliminary *Bla*st search, which may indicate they are transferable (data not shown). Our findings corroborate that *mcr*-1 and *mcr*-3 genes are frequently isolated in *E. coli* from pigs and humans in Vietnam [26,29,37,38]. Similar observations have been reported for *E. coli* originating from chickens in Vietnam [3]. The frequent finding of colistin and quinolone-resistance in ESBL-producing *E. coli* in pigs may pose a food safety concern and the level of transfer from pigs should be determined in order to assess whether they are implicated in human disease. 

A few discrepancies were observed in the genotypic and phenotypic resistance. For instance, the lack of colistin resistance in the human isolate Ec309 could be attributed to the absence of any *mcr* genes in the genome of this isolate, which only harbors the *pmr*B mutation. Similarly, the presence of *qnr*S1 alone was not enough to yield resistance to nalixidic acid and ciprofloxacin in the strains lacking the required mutations on the quinolone resistance-determining region.

Commensal *E. coli* are less studied than pathogenic *E. coli*; they are usually genetically distinct [23] and often harbor various resistance genotypes with the potential to be horizontally transferred to bacterial pathogens through the mobile genetic elements they contain, therefore representing a public health risk [26]. Opportunistic infections caused by the antimicrobial-resistant commensals, themselves, may also occur. Our data show that multiple resistance genes, along with ESBL genes, *mcr* genes, and quinolone resistance genes, are harbored across diverse *E. coli* lineages of predominantly commensals types and are epidemiologically unrelated. Similar observations of high frequency drug resistance in commensal *E. coli* have been reported in ESBL strains in Poland [39]. The public health relevance of our commensal *E. coli* is further emphasized by the fact that they were predominately MDR [40], which is in agreement with previous studies documenting that ESBL-producing *E. coli*, whether commensal or pathogenic, are MDR [3,29,41,42]. Fifty-five of the farms studied bought piglets from other farms or pig companies, and the ESBL-producing *E. coli* found in mainly finisher pigs may have originated from such sources. Even in farms with a low use of antimicrobials, MDR *E. coli* are frequent probably because the strains circulating in the production system have been subjected to high selective pressure for a long time. Thus, weaned piglets from Danish pig farms not using antimicrobials have been shown to contain a high diversity of MDR commensal *E. coli* [23]. Various studies have documented the frequent use of third generation cephalosporin, quinolones, and colistin in humans and livestock in Vietnam [7,9,35,43], and that a high number of such antimicrobial products are easily accessible over the counter often without the need for medical and veterinary prescriptions [44,45]. However, it remains to be determined to what extent the high levels of antimicrobial resistance at our pig farms were associated with actual antimicrobial use or other driving factors.

There is an association between metals resistance and the occurrence of antimicrobial resistance, where exposure to metals such as zinc oxide is often applied to control diarrhea in weaning pigs and copper added to feed for growth promotion can co-select for antimicrobial bacterial resistance [46,47,48]. The MDR ESBL-producing *E. coli* characterized in this study contained a number of metal resistance genes, which may be associated with the high level of antimicrobial resistance. Twenty out of 74 ESBL-producing *E. coli* isolates carried heavy metal resistance genes and ESBL genes located on plasmid(s). Resistance to metals and antimicrobials may be associated as these are frequently located on the same mobile elements [49]. The abundance of heavy metal resistance genes on plasmids found in this study may contribute to the dissemination of ESBL genes. Unfortunately, we do not have sufficient data on the antimicrobial and metal use from the pig farms to evaluate to what extent the antimicrobial resistance found was associated with antimicrobial and metal exposures.

## 4. Materials and Methods

### 4.1. Collection of Fecal Samples

Fecal samples were collected from pigs and workers at 116 pig farms in Bac Ninh Province in Northern Vietnam. The farms varied in size and were randomly selected from a list of pig farms provided by veterinary authorities in the province. A total of 116 composite pig manure samples and 94 fecal samples from healthy farm workers were collected. A sample was collected from the top of fecal piles on the floor with a spoon from three locations within each pig pen, after which samples were pooled. A composite fecal sample with a weight between 100 g and 200 g which included feces from the different pig pens was collected in each farm. A maximum of three pig pens per farm were sampled. Each pen was randomly chosen to represent the pig type (sow, piglet, and finisher) in a total of 61 farms that produced their own piglets and raised them until slaughter. An approximately 20 g fecal sample was self-collected by one worker during defecation using sterile gloves on the same day as the pigs were sampled. Fecal samples were placed in labelled sterile plastic bags and immediately transported to the laboratory at the National Institute of Veterinary Research in Hanoi, at which they were analyzed the same day. Both farm owner and farm workers gave consent orally and understood that he/she could withdraw from the study at any time and would be anonymous in the reporting of the results. The study protocol was approved by the Ethical Committee of the National Institute of Nutrition in Hanoi (certificate number 04/VDD-QLKH).

### 4.2. Sampling and Isolation of E. coli

Ten grams of fecal sample was mixed with 90 mL of Peptone Buffered Saline containing 0.1% peptone (Himedia, Mumbai, India) and 0.85% NaCl in a sterilized plastic bag, and was homogenized in a stomacher. One 10-µL loop of the dilution was spread onto MacConkey agar (Merck, Darmstadt, Germany) plates with and without 2 mg/L of cefotaxime (breakpoint concentration according to EUCAST [50]) and was incubated at 37 °C for 24 h for the selection of *E. coli* colonies [51]. Up to five presumptive *E. coli* colonies (red, smooth, round, >2.5 mm diameter) that showed bacterial growth were selected from each plate with and without cefotaxime (Sigma Aldrich, St Louis, MO, USA) [51]. All colonies were selected if less than five colonies appeared on the MacConkey agar with 2 mg cefotaxime. Confirmatory biochemical testing for *E. coli* included glucose (+), lactose (+), gas (+), H_2_S (−), indole (+), urease (−), Voges–Proskauer (−), methyl red (+), and citrate (+) [51]. *E. coli* ATCC 25,922 was used as reference strain for quality control. Confirmed isolates were purified on blood agar plates and stored at −80 °C in Eppendorf tubes containing Brain Heart Infusion broth (CM1135; Oxoid, Basingstoke, UK) with 10% glycerol.

### 4.3. Antimicrobial Susceptibility Testing

One colony confirmed as *E. coli* was randomly selected on agar plates with and without cefotaxime per fecal sample and was tested for susceptibility to: ampicillin (AMP, 10 µg), gentamycin (GEN, 10 µg), trimethoprim (TMP, 5 µg), tetracycline (TET, 30 µg), streptomycin (STR, 10 µg), nalidixic acid (NAL, 30 µg), sulfonamides (SUL, 300 µg), ciprofloxacin (CIP, 5 µg), ceftriaxone (CRO, 30 µg), cefoxitin (FOX, 30 µg), ceftiofur (EFT, 30 µg), and amoxicillin-clavulanic (AMC, 30 µg) by the Kirby–Bauer disc diffusion test according to the CLSI standard procedure [52]. Antimicrobial discs were from the Oxoid company (Basingstoke, UK). Isolates which were resistant to CRO were also tested for ESBL production by the modified double-disk synergy test (amoxicillin/clavulanic acid, 20/10 µg) [53]. The interpretation of inhibition zones was performed according to CLSI criteria and the isolates showing intermediate resistance were categorized as susceptible to avoid overestimation of resistance. Micro-broth dilution assay was done to confirm colistin (COL) resistance, using dilutions of colistin sulphate from 32 µg/mL to 0.25 µg/mL (Sigma Aldrich, St Louis, MO, USA). *E. coli* ATCC 25,922 was used as a control strain (MIC of colistin: 0.25–2 µg/mL). Multidrug resistance (MDR) was defined when an isolate was not susceptible to at least one antimicrobial in at least three of the tested antimicrobial classes [40]. Prob.test in R studio was used to compare differences between the AMR proportion of two populations.

### 4.4. Whole-Genome Sequencing and Sequence Analysis

Two criteria were used to select confirmed ESBL-producing *E. coli* isolates from MacConkey agar plates with cefotaxime for whole-genome sequencing (WGS). One human and one pig ESBL-producing *E. coli* from 26 farms that had ESBL-producing isolates in the fecal samples from both hosts were selected (*n* = 52). ESBL-producing *E. coli* isolated from pigs and humans in the remaining farms were selected for WGS if they had different resistance profiles compared to the those from the 26 farms described above. Thus, a total of 74 ESBL-producing *E. coli* isolates, including 43 pig isolates and 31 human isolates, were selected. There was less human *E. coli* compared to the pig isolates because some human fecal specimens did not yield any *E. coli* colonies on the MacConkey agar plates containing cefotaxime. DNA was extracted by using an automated Maxwell DNA extraction system following the manufacturer’s instruction (Promega Maxwell RSC, Maldison, WI, USA). The concentration of the extracted DNA was determined using NanoDrop. The DNA quality was checked following electrophoresis in a 1% agarose gel. The genomic DNA was sequenced on an Illumina pair-end sequencing platform using the Illumina Nextera XT and MiSeq reagent kit v.3 protocol. The paired-end raw reads were assembled using Spades 3.9 [54] and were quality checked using Quast [55]. The sequence reads have been submitted to the European Nucleotide Archive with the accession number PRJEB37980. Assembled sequences were analyzed using tools from the servers of the Center for Genomic Epidemiology (CGE) (https://cge.cbs.dtu.dk/services/ (accessed on 15 June 2020) for multi-locus sequence types using MLST 2.0. Antimicrobial resistance genes using ResFinder 4. Virulence determinants were assessed in the genomes using VirulenceFinder 2.0 from CGE, with default settings coupled with the BLAST algorithms through MyDbFinder (https://cge.cbs.dtu.dk/services/ (accessed on 15 June 2020) using new curated databases of Extra-intestinal pathogenic *E. coli* genes (ExPEC) and *eae* genes for the attaching and effacing of *E. coli* (AEEC) [56]. The presumptive classification of *E. coli* isolates used two definitions, which classify isolates as (1) ExPEC_JJ_ if positive for two or more of *papAH* and/or *papC* (P fimbriae), *sfa/focDE* (S and F1C fimbriae), *afa/draBC* (Dr-binding adhesins), *iutA* (aerobactin siderophore system), and kpsM II (group 2 capsules) [18], and as (2) uropathogenic *E. coli* (UPEC_HM_) if positive for two or more of *chuA* (heme uptake), *fyuA* (yersiniabactin siderophore system), *vat* (vacuolating toxin), and *yfcV* (adhesin) [19].

Metal resistance genes encoding resistance to copper, cobalt, mercury, zinc, cadmium, magnesium, and chromium were analyzed through the subsystem annotation in RAST [57]. Resistance to tellurite was detected by using *BLA*STn with the tellurite resistance genes tehA_NC_000913.3, tehB_M74072.1, and telluECs2035_EU901290.1. The detergent-resistant phospholipase A and pldA (NC_003198.1) as well as the quaternary ammonium compound efflux qacEdelta (NG_048042.1) were also searched for in the genomes using *Bla*st.

The presence of plasmid replicons was determined using PlasmidFinder and the serotypes were determined using SerotypeFinder 2.0. Strains, in which the O antigen was not detected in the initial analysis, were subjected to a *BLA*ST alignment using the O serotype database for confirmation. Enterobase (http://enterobase.warwick.ac.uk/ (accessed on 20 June 2020) was used to determine the phylogroups of the strains and to confirm both the serotypes and MLST findings. A phylogenetic analysis was performed with the 74 isolates using the pipeline CSI phylogeny 1.4 tool from the servers of the Center for Genomic Epidemiology (CGE) [58] with automate default settings using *E. coli* K12 substrain.MG1655 (accession number U00096) as reference. This built a consensus core-genome tree and generated a Newick tree file, along with the multiple alignment of the core-genomes’ SNPs. The obtained tree was annotated in Interactive Tree Of Life iTOL v3 [59] and was interpreted along with the SNP (single nucleotide polymorphism) values between the genomes. Prob.test in R studio was used to compare the difference distribution between the pig and human isolates in each phylogenic cluster.

## 5. Conclusions

The results from our genetic diversity and phylogenic analysis of ESBL-producing *E. coli* showed that ESBL-producing *E. coli* from pigs were different from the ESBL-producing *E. coli* found in pig farm workers on the same farms. The highly heterogeneous population of commensal MDR ESBL-producing *E. coli* carrying plasmid-associated colistin and quinolone resistance genes, as well as carrying detergent and metal resistance genes, represent a potential source and possible hotspot for the horizontal transmission of antimicrobial resistance. Our findings confirm that the prevalence of ESBL-producing *E. coli* in people and animals is high in Vietnam. A main conclusion is that the direct contact with pigs may not be a likely contributor to the carriage of ESBL *E. coli* in farm workers. This does not rule out that pig strains can be transmitted to humans via the food chain and that the role of food from animal origin in the transmission of ESBL-producing *E. coli* to humans should be assessed. Moreover, actual antimicrobial use and other pig farm management factors impacting the emergence and maintenance of the antimicrobial resistance in pigs remains to be determined.

## Figures and Tables

**Table 1:** Prevalence of antimicrobial resistance in *E. coli* isolated from pigs and pig farm workers.

Antimicrobial	Farm Worker Feces (N = 94)	Pig Manure (N = 116)	*p-*Value
	Number of Resistant Isolates *n* (%)	Number of Resistant Isolates *n* (%)	
Ampicillin (AMP)	68 (72.3)	100 (86.2)	0.02
Gentamycin (GEN)	11 (11.7)	34 (29.3)	0.003
Trimethoprim (TMP)	61 (64.9)	84 (72.4)	0.30
Tetracycline (TET)	68 (72.3)	98 (84.5)	0.04
Streptomycin (STR)	40 (42.6)	65 (56.0)	0.07
Nalidixic acid (NAL)	25 (26.6)	32 (27.6)	0.99
Sulphonamide (SUL)	63 (67.0)	96 (82.8)	0.01
Ciprofloxacin (CIP)	11 (11.7)	17 (14.7)	0.67
Ceftriaxone (CRO)	13 (13.8)	1 (0.9)	0.0005
Cefoxitin (FOX)	4 (4.3)	0 (0.0)	
Amoxicillin-clavulanic acid (AMC)	4 (4.3)	3 (2.6)	
Ceftiofur (EFT)	13 (13.8)	1 (0.9)	0.0005
Colistin (COL)	14 (14.9)	44 (37.9)	0.003

**Table 2:** Mutations in the quinolone resistance-determining region (QRDR) of ESSBL-producing *E. coli* from pigs and pig farm workers.

Topoisomearase Gene	Mutation Region and Amino Acid Change	Number of Pig Isolates (*n*) (N = 43)	Number of Human Isolates (*n*) (N = 31)
*gyr*A	Ser83→Leu	19	17
	Asp87→Ans	9	6
	Asp87→Ala		1
	Asp87→Gly	1	
	Asp87→His		1
	Asp87→Tyr		2
*par*C	Ser80→Ile	11	10
	Ser56→Thr	3	
	Ser57→Thr	1	
*par*E	Ser458→Ala	1	4
	Iso335→Thr		1
	Leu416→Phe		1

Abbreviation: Ser, Serine; Leu, Lecine; Asp, Aspartic acid; Ala, Alanin; Ans, Asparagine; Gly, Glycine; His, Histadine; Tyr, Tyrosine; Ile, Isoleucine; Thr, Threonine; and Phe, Phenilalaniene.

**Table 3:** Genomic characterization of ESBL-producing *E. coli* isolated from pig farm workers and pigs.

Strain ID	MLST	Genome Size	GC%	N50	Phylogroup	Serotype	Virulence Genes	Plasmid Replicons
Ec_1 ^p^ *	ST5855	4,548,521	51.12	21,866	A	H38	*ompT, terC*	IncY
Ec_3 ^p^	ST4956	4,647,525	51.1	23,906	B1	H37	*lpfA, terC*	ND
Ec_6 ^p^	ST5870	4,899,146	51.03	50,792	A	O147:H40	*astA, terC, traT*	IncFIA (HI1), IncFIB (K), IncFII, IncN, IncX1
Ec_7 ^p^	ST877	4,728,224	50.94	29,266	B1	O45:H10	*iss, lpfA, ompT, terC, traT,*	IncFII, IncR
Ec_9 ^p^	ST10	4,873,762	50.71	71,377	A	H32	*terC*	IncFIA (HI1), IncFIB (K), IncHI1A, IncHI1B (R27)
Ec_21 ^p^	ST10	4,770,784	50.89	72,227	A	O45:H45	*kpsE, kpsMII, terC*	IncFIA (HI1), IncFIB (K), IncX1
Ec_219 ^h*^	ST1163	4,905,795	51.02	73,212	G	H23	*iss, lpfA, senB, chuA, neuC, ompT, terC, traT*	Col156, IncFIA, IncFIB (AP001918), IncFII
Ec_309 ^p^	ST10	4,820,803	50.9	43,099	A	H32	*iss, terC*	ND
Ec_335 ^h^	ST394	5,103,214	50.52	72,314	D	O17/O77:H18	*aap, air, astA, eilA, lpfA, chuA, kpsE, kpsMII_K1, neuC, terC, traT*	IncFII, IncFII (pHN7A8)
Ec_314 ^p^	ST2170	5,061,254	50.7	43,068	A	H51	*cma, iss, lpfA, fyuA, hlyF, irp2, iucC, iutA, ompT, sitA, terC, traT*	IncFIB (AP001918), IncFIB (Plf82-PhagePlasmid), IncFIC (FII), IncI1-I (gamma)
Ec_340 ^h^	ST345	4,880,303	50.61	82,846	B1	O8:H21 (53)	*lpfA, terC*	IncI1, IncY
Ec_116 ^p^	ST3588	4,866,794	50.99	23,092	ND	O8:H7	*iroN, iss, lpfA, mchF, tsh, cvaC, etsC, hlyF, iucC, iutA, ompT, sitA, terC, traT*	IncFIB (AP001918), IncFIC (FII), IncFII (pHN7A8), IncN
Ec_224 ^h^	ST196	5,127,497	50.46	85,977	B1	O8:H7	*iroN, iss, lpfA, hlyF, ompT, sitA, terC, traT*	IncFIA (HI1), IncFIB (AP001918), IncFII (pCoo), IncHI1A, IncHI1B (R27), IncI2, p0111
Ec_123 ^p^	ST218	4,902,464	51	38,282	A	H23	*TerC, TraT*	IncFIA (HI1), IncFIB (K), IncFII, IncN, IncX1
Ec_129 ^p^	ST195	4,641,276	50.92	27,405	ND	H4	*lpfA, terC*	ND
Ec_228 ^h^	ST68	4,995,853	50.73	68,496	D	H6	*air, eilA, iss, lpfA, chuA, kpsE, kpsMII, sitA, terC,*	IncFIB (K)
Ec_130 ^p^	ST48	4,963,599	50.75	51,687	B1	O15:H11	*astA, ompT, terC*	IncN, IncX1, p0111
Ec_131 ^p^	ST515	5,015,450	50.61	73,216	B1	H12	*fyuA, irp2, terC*	IncFIA (HI1), IncHI1A, IncHI1B (R27)
Ec_274 ^p^	ST278	4,936,780	50.61	61,512	B1	H7	*fim41A, lpfA, terC*	IncFIB (pB171), IncFII, IncX1
Ec_230 ^h^	ST351	5,181,680	50.5	68,811	B1	O18:H7	*cma, iroN, iss, cvaC, etsC, hlyF, ompT, papC, sitA, terC*	IncFIA (HI1), IncFIB (AP001918), IncFII, IncHI1A, IncHI1B (R27)
Ec_400 ^h^	ST156	5,241,723	50.5	42,932	A	O76:H45	*cma, astA, hlyF, hra, iucC, iutA, ompT, papC, sitA, terC, traT*	IncFIB (AP001918), IncFIC (FII)
Ec_361 ^p^	ST10	5,207,190	50.7	75,974	A	O29:H10	*astA, sitA, terC*	IncHI2, IncHI2A, IncQ1, IncY
Ec_405 ^h^	ST1286	5,984,169	50.34	26,744	B1	O71:H32	*aap, astA, iha, mchB, mchC, mchF, fyuA, irp2, terC, traT*	IncB/O/K/Z, IncFII, IncFII (pHN7A8)
Ec_381 ^p^	ST29	5,102,316	50.59	73,837	B1	O123/186:H11	*astA, cif, eae, efa1, espA, espB, espJ, iha, iss, katP, lpfA, nleA, nleB, nleC, sepA, tir, cea, iucC, iutA, ompT, terC, traT, fyuA, irp2*	IncFIB (AP001918), IncFII (pHN7A8), IncHI2, IncHI2A
Ec_410 ^h^	ST2170	5,023,174	50.9	32,449	B1	H51	*cma, iss, lpfA, fyuA, hlyF, irp2, iucC, iutA, ompT, sitA, terC, traT*	IncFIB (AP001918), IncFIB (pLF82), IncFIC (FII), IncI1
Ec_388 ^p^	ST5229	4,859,334	50.86	30,295	ND	O177:H34	*astA, lpfA, fyuA, irp2, iucC, iutA, papC, sitA, terA, traT*	IncHI2, IncFIB (AP001918), IncFII (pHN7A8), IncN
Ec_155 ^p^	ST165	4,879,514	50.98	37,635	ND	H26	*terC*	IncQ1, IncR, IncX1, IncY
Ec_170 ^h^	ST1147	5,104,121	50.7	91,960	B1	H35	*aap, aatA, iha, iss, lpfA, fyuA, irp2, terC, traT*	IncFIB (AP001918), IncFII (29)
Ec_278 ^p^	ST744	4,925,781	50.7	103,452	A	O162/89:H9	*cma, cvaC, hlyF, iucC, iutA, ompT, sitA, terC, traT*	IncFIB (AP001918), IncFIC (FII), IncN, IncQ1, IncX4
Ec_172 ^h^	ST69	5,391,031	50.77	72,381	D	O15:H18	*air, eilA, iha, iss, lpfA, sat, senB, chuA, fyuA, irp2, iucC, iutA, kpsE, kpsMII_K52, ompT, papA_fsiA_F16, papC, sitA, terC, traT*	Col156, IncB/O/K/Z, IncFIB (AP001918)
Ec_162 ^p^	ST2170	4,901,850	50.7	65,441	B1	O78:H51	*cma, iss, lpfA, fyuA, hlyF, irp2, iucC, iutA, ompT, sitA, terC, traT*	IncFIB (AP001918), IncFIC (FII), IncI2
Ec_166 ^p^	ST2954	4,803,218	50.87	19,954	ND	O160:H9	*air, chuA, terC*	IncFIB (pLF82), p0111
Ec_232 ^h^	ST167	4,956,803	50.84	70,019	A	O-89 (162):H10	*fyuA, irp2, terC, traT, sitA*	Col (BS512), IncFIA, IncFIB (AP001918), IncFII
Ec_168 ^p^	ST2705	4,726,759	50.8	35,199	A	H10	*ompT, TerC*	p0111
Ec_179 ^h^	ST5044	4,523,830	50.79	93,960	B1	H29	*terC*	ND
Ec_283 ^p^	ST1011	5,130,567	50.55	61,007	D	O8:H16	*air, cma, eilA, iroB, iss, chuA, cvaC, hlyF, iucC, iutA, ompT, sitA, terC, traT*	IncFIB (AP001918), IncFIB (pLF82), IncFIC (FII), IncX4
Ec_53 ^p^	ST6775	4,825,481	50.8	26,621	B1	O65:H49	*iss, terC*	IncHI2, IncHI2A, IncX1
Ec_236 ^h^	ST1294	4,511,999	50.78	126,228	A	O9:H30	*terC*	ND
Ec_57 ^p^	ST165	5,115,907	50.56	52,960	A	H27	*terC*	IncHI2, IncHI2A, IncQ1, IncR, IncX1
Ec_292 ^p^	ST1121	4,893,986	50.7	83,326	A	H48	*AstA, lpfA, terC*	IncN, IncY
Ec_416 ^p^	ST10	4,680,174	50.7	97,966	A	O25:H32	*terC*	IncFIB (K)
Ec_480 ^h^	ST34	4,650,258	50.92	60,644	A	H30	*terC*	IncFIA (HI1), IncFIB (K)
Ec_426 ^p^	ST2935	4,875,048	50.86	54,178	A	H32	*astA, terC*	IncP1, IncQ1, IncX1, IncY
Ec_488 ^h^	ST484	4,877,138	50.57	68,561	A	H4	*aap, astA, iss, kpsE, kpsMII, ompT, terC, traT*	IncFIC (FII), IncFII (pCoo)
Ec_431 ^p^	ST226	4,694,058	51	45,154	A	O15:H10	*fyuA, irp2, terC*	ND
Ec_489 ^h^	ST34	5,015,072	50.6	59,062	A	O68 (62):H30	*astA, TerC*	IncFIA (HI1), IncFIB (K), IncHI1A, IncHI1B (R27), IncR, IncX1
Ec_434 ^p^	ST641	4,959,009	50.7	29,769	B1	H21	*astA, lpfA, terC*	IncHI2, IncHI2A
Ec_495 ^h^	ST1722	5,038,475	50.7	83,575	F	H24	*air, eclb, eilA, iss, lpfA, chuA, fyuA, irp2, kpsE, sitA, terC, traT*	Col (IMGS31), Col156, IncFIA, IncFIB (AP001918), IncFII
Ec_297 ^p^	ST410	5,069,502	50.6	48,156	C	O33:H26	*iss, lpfA, ompT, TerC, traT*	IncFIA (HI1), IncFIB (AP001918), IncFII (pHN7A8), IncHI1A, IncHI1B (R27), IncI1
Ec_60 ^p^	ST515	5,084,460	50.9	65,609	B1	O128:H12	*fyuA, irp2, terC, traT*	IncFIB (AP001918)
Ec_84 ^h^	ST48	4,947,358	50.76	72,323	A	O20 (137):H45	*cma, iroN, iss, katP, mchF, cvaC, hlyF, iucC, iutA, ompT, sitA, terC, traT*	IncFIB (AP001918), IncP1
Ec_63 ^p^	ST515	4,812,948	50.9	55,529	B1	O128:H12	*fyuA, irp2, terC*	Col440II
Ec_89 ^h^	ST2040	4,932,400	50.7	69,363	A	O159:H20	*astA, lpsA, cib, sitA, terC*	IncB/O/K/Z, IncFII (pCoo)
Ec_67 ^p^	ST6214	5,112,157	50.9	56,813	B1	O162/89:H10	*iss, nfaE, afaA, afaB, afaC, afaD, afaE, fyuA, irp2, iucC, iutA, sitA, TerC, TraT*	IncFIB (AP001918), IncFII (pRSB107)
Ec_93 ^h^	ST162	5,050,320	50.6	72,932	B1	O8:H21	*astA, iss, lpfA, hlyF, hra, iucC, iutA, ompT, papA-NEW, papC, sitA, terC, traT*	IncFIB (AP001918), IncFIC (FII)
Ec_255 ^h^	ST1722	4,823,623	50.62	78,611	F	O1:H25	*air, astA, eilA, iss, lpfA, chuA, hra, kpsE, terC, yfcV*	IncQ1
Ec_98 ^h^	ST7153	4,746,746	50.75	49,123	A	O148:H30	*astA, terC, traT*	IncFIA (HI1), IncFIB (K), IncX1, IncY
Ec_80 ^p^	ST515	4,962,369	50.9	38,744	B1	O128:H12	*fyuA, irp2, terC, traT*	IncFIB (AP001918), p0111
Ec_271 ^h^	ST1196	4,931,876	50.84	129,873	B1	O29:H8	*cma, lpfA, cea, cvaC, hlyF, iucC, iutA, ompT, sitA, terC, traT*	IncFIB (AP001918), IncFIC (FII)
Ec_81 ^p^	ST877	4,744,811	50.8	59,541	B1	O28ac/O42:H32	*lpfA, ompT, TerC*	IncFIB (AP001918), IncFIA (HI1), IncFIB (K), Inc1
Ec_447 ^p^	ST4956	4,805,635	50.9	54,620	B1	O156:H37	*lpfA, terC, traT*	IncHI2, IncFIB (AP001918), IncFIC (FII)
Ec_498 ^h^	ST10	4,743,370	50.91	77,783	A	O9 (89,162):H9	*sitA, terC, traT*	IncFIA, IncFIB (AP001918), IncFII
Ec_83 ^p^	ST6018	4,752,755	50.79	69,914	A	O8:H11	*ompT, terC*	IncX1, p0111
Ec_442 ^p^	ST101	5,046,847	50.26	71,098	B1	O118:H21	*cma, iss, lpfA, cvaC, hlyF, iucC, iutA, ompT, sitA, terC, traT*	IncFIB (AP001918), IncFIC (FII), IncFII (29), IncI2
Ec_452 ^p^	ST515	4,878,968	51	42,049	B1	O128ac:H12	*fyuA, irp2, terC, traT*	IncFIB (AP001918), IncX4
Ec_500 ^h^	ST2040	4,895,059	50.77	26,675	A	O159:H20	*astA, lpfA, cib, sitA, terC*	IncB/O/K/Z, IncFII (pCoo)
Ec_457 ^p^	ST1121	4,935,661	50.57	131,601	A	H48	*lpfA, terC*	IncX4, IncY
Ec_503 ^h^	ST192	4,868,610	50.73	79,109	B1	O124 (164):H34	*iss, lpfA, ompT, terC*	IncX2, p0111
Ec_511 ^h^	ST38	5,198,810	50.66	73,146	D	O1:H15	*air, iha, iss, sat, senB, chuA, fyuA, hra, irp2, iucC, iutA, kpsEkpsMII_K5, papA_F43, terC, traT, kpsE*	Col156, IncFIA, IncFIB (AP001918), IncFII (pRSB107)
Ec_467 ^p^	ST4238	4,663,515	50.71	92,493	A	H2	*capU, terC*	IncFIB (K)
Ec_514 ^h^	ST38	5,084,740	50.7	54,474	D	O86:H18	*air, astA, eilA, iss, afaD, chuA, fyuA, irp2, kpsE, kpsMII_K5, terC*	IncL/M (pOXA-48)
Ec_472 ^p^	ST877	4,948,850	50.7	72,864	B1	H10	*lpfA, ompT, TerC, traT*	IncFII (pCoo), IncI1, IncX1
Ec_517 ^h^	ST1141	5,068,761	50.82	55,942	B1	O13:H11	*astA, terC, traT*	Col (BS512), Col (IRGK), IncFIB (K), IncR, IncY
Ec_519 ^h^	ST6253	5,382,277	50.8	35,706	D	H15	*aap, air, astA, capU, eatA, eilA, chuA, kpsE, kpsMII_K5, terC, traT*	IncFII

* P: Pig isolates; h: Human isolates.

## Data Availability

All data supporting this study are available in the supplementary materials. Moreover, the genome sequence reads have been deposited to the European Nucleotide Archive and publicly available with the accession number PRJEB37980.

## Supplementary Materials

The following are available online at www.mdpi.com/article/10.3390/antibiotics10101165/s1, Table S1: Resistance profiles of indicator *E. coli* isolated from pigs and farm workers; Table S2: Resistance profiles of ESBL-producing *E. coli* isolated from pigs and farm workers; Table S3: Phenotypic and genotypic characteristic of ESBL-producing *E. coli* isolated from pigs and farm workers; Table S4: Matrix of main SNPs of ESBL-producing *E. coli* isolated from pigs and farm workers; and Table S5: Metal and detergent resistance genes of ESBL-producing *E. coli* isolated from pigs and farm workers.
